# New Dietary Trends—Meal Kit Delivery Services as a Source of Nutrients: A Scoping Review

**DOI:** 10.3390/nu17071154

**Published:** 2025-03-26

**Authors:** Dominika Patrycja Dobiecka, Renata Markiewicz-Żukowska, Katarzyna Socha, Sylwia Katarzyna Naliwajko

**Affiliations:** Department of Bromatology, Faculty of Pharmacy with the Division of Laboratory Medicine, Medical University of Białystok, 15-222 Białystok, Poland; renata.markiewicz@umb.edu.pl (R.M.-Ż.); katarzyna.socha@umb.edu.pl (K.S.); sylwia.naliwajko@umb.edu.pl (S.K.N.)

**Keywords:** meal kit, meal kit delivery, meal kit delivery services, nutrition, food insecurity

## Abstract

Producers and distributors of meal kits promote their products by emphasising customisation and a health-conscious approach to eating. Consumers of these services expect that, for an appropriate fee, they will receive a nutritious and flavourful meal, tailored to their individual needs and conveniently delivered to their homes. This raises the question of whether meal kit companies meet these expectations and whether their products are prepared with the level of care claimed in their advertisements. This scoping review aims to present available evidence that offers insight into the nutritional content, safety, and acceptability of meal kit delivery services. In this context, we have identified 15 publications. To the best of our knowledge, this is the first scoping review to focus specifically on meal kits. The findings highlight the significant potential of meal kits and may contribute to efforts to enhance their quality. Available research on meal kit delivery services was conducted using calculation methods. Nutritional value studies involving analytical methods and intervention studies are necessary in order to expand the understanding of the potential of meal kits role in whole-food nutrition.

## 1. Introduction

Due to the fast pace of life and work, more and more people find they do not have time to prepare healthy and nutritious meals. They take advantage of catering companies that deliver ready-made kits with fresh, pre-measured products and recipes to quickly prepare of healthy meals [1]. In some countries, such as Poland, ready-to-eat meals packaged in convenient containers are also very popular.

The basic range of products most companies provide includes standard diets with options to choose the calorie count and number of meals. Diets for athletes, vegetarians, people with allergies, and those dealing with certain medical conditions are also in high demand. Companies also offer the option to eliminate poorly tolerated or disliked food products from the diet, as well as the ability to select meals from a wide menu [2,3]. In 2022, the meal kit delivery services market worldwide was valued at USD 20.54 billion and is expected to grow at a compound annual growth rate (CAGR) of 15.3% from 2023 to 2030 [4]. According to a 2024 report, 13–14% of Poles use catering services, and the meal kit delivery market in 2023 exceeded PLN 3 billion only in Poland [5]. Consumers place orders online either in the form of a subscription or a single service.

Meal kit delivery services have gained many supporters, and companies providing said services are becoming more and more popular [6]. A significant increase in the interest in ‘meal delivery diets’ was observed during the COVID-19 pandemic [7]. Undoubtedly, one of the main reasons for opting for delivered meals was the curb on leaving home to shop [8]. While home isolation led to weight gain for a significant portion of the population, it also prompted positive dietary changes for others, fostering greater awareness of food choices. This shift may have contributed to the increasing interest in this type of nutrition [9,10,11,12]. Receiving ready-made bags of products and simple recipes satisfied most of the customers. According to the outcome of the research conducted in 2021, the largest meal delivery providers in the USA enjoyed a 49% increase in the number of customers in 2020, while concurrently doubling the sales of meal kit sets compared with the previous year [13]. Recent market research predicts that, by 2032, the size of the meal kit delivery service market will have increased by as much as tenfold as compared with that in 2020 [14].

Meal kits appear to be a healthier alternative to meals delivered from traditional caterers or convenient ready-to-eat food bought in supermarkets [15]. The primary advantage is that consumers have full transparency regarding the ingredients in their prepared meals and can assess their freshness. Meal kits primarily include fresh produce, such as fruits, vegetables, eggs, meat, and fish. However, consumers are typically required to provide certain essential ingredients, such as basic spices (e.g., salt and pepper) and cooking oil [16].

People using meal kits declare that having switched to that type of diet has enabled them to significantly reduce their food waste [17,18,19]. Moreover, studies show that meal kits are more beneficial to the environment than meals sold in stores, as evidenced by the lower greenhouse gas emissions resulting from the use of reusable containers, an optimised supply chain delivering meals directly to customers, and the previously mentioned reduction in food waste [20,21].

Although the nutrient content of meal kits has been previously studied, all available findings rely on calculation methods, rather than direct laboratory analysis. To date, no studies have evaluated the nutrient composition of meal kits using laboratory-based methods. In previous research, Researcher et al. assessed the protein content of 45 daily food rations, each consisting of three to five meals with a total caloric value of 2000 kcal, using the Kjeldahl method. The analysis included meals adhering to the principles of the DASH diet, the low-carbohydrate diet, and dietary guidelines for patients with Hashimoto’s disease. The protein content of the daily rations ranged from 48.4 g to 208.8 g per 2000 kcal. Additionally, 24% of the analysed rations contained more protein than declared by the manufacturer (exceeding 120% of the stated value), while 11.1% contained less than 80% of the declared protein content. Notably, more than half (56%) of the daily food rations qualified as high-protein diets, with protein contributing over 20% of total energy intake [22].

Due to the increasing popularity of take-away meal kits, it seems necessary to systematise the research available on the subject, which is still scarce. The compiled literature data will allow for an assessment of that type of nutrition from both nutritional and qualitative perspectives. Furthermore, this review will support the foreground knowledge on the possibility of using meal kits in a daily diet in line with healthy eating principles.

The purpose of this scoping review is to present available evidence that provides for the insight into the nutritional content, safety, and acceptability of meal kit delivery services.

## 2. Materials and Methods

### 2.1. Search Strategy

The presented scoping review was prepared in accordance with the Preferred Reporting Items for Systematic Reviews and Meta-Analyses (PRISMA) guidelines [23]. Four databases were used for gathering the literature: PubMed, Web of Science, Scopus, and the Cochrane Library. The data collected for the purpose of this study date back to June 2024–July 2024 and cover papers from June 2014 to July 2024. The database review searched for terms including ‘meal kit’ or ‘meal kit services’ or ‘meal kit delivery services’.

### 2.2. Data Extraction

**Figure 1:**
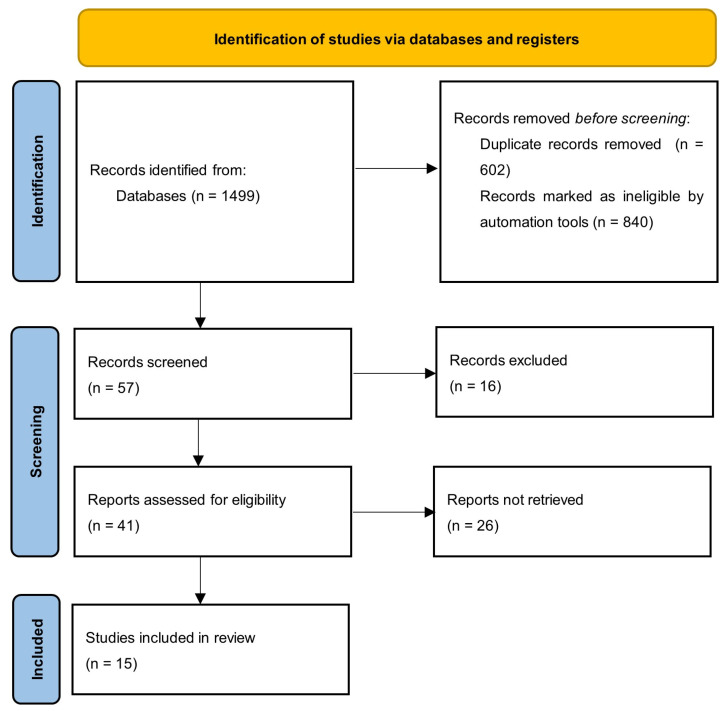
Flow diagram for scoping review.

The search initially found 1499 records that were collected and imported into EndNote 21 (Clarivate Analytics). After having removed duplicates, the number decreased to 897. Given the adopted criteria, 840 papers were excluded. After a detailed review of the topics and abstracts, 41 papers were considered to be of potential interest. The above papers were subjected to full-text evaluation, which resulted in the inclusion of 15 papers in this study. The search strategy is shown in Figure 1. The characteristics of the included studies are summarised in Table 1, Table 2, Table 3 and Table 4. Four of them were descriptive research, three were cross-sectional, three were pilot studies, two were qualitative research, one was longitudinal, one was implementation research, and one was evaluative research. None of the authors declared any conflicts of interest.

## 3. Results

### 3.1. Assessment of Nutrient Content

Studies examining the nutrient contents of meal kits were analysed. The review included five descriptive studies focusing on meal kits available in Australia, with no studies identified from other geographical regions. The collected data provide information on energy, macronutrient, fibre, vitamin, and mineral contents, comparing these values with the dietary standards of the Australian and New Zealand populations. The overall findings on the nutrient composition of meal kit portions are summarised in Table 1 and Table 2. In all studies, nutrient content was assessed using computational methods. The analysis of the meal kits showed that most provided significant amounts of fat (up to 59.6% of energy) [24,25,26], including saturated fat (11% of energy) [25], and more than 2000 mg of sodium, to exceed the total recommended dietary intake for those elements [24,25,26,27,28]. Additionally, manufacturers recommended adding extra amounts of sodium to the meals to enhance their flavour. Excessive sodium intake in the diet can contribute to the development of hypertension, heart disease, and stroke, metabolic disorders, kidney diseases, and increase the risk of stomach cancer [29,30,31]. In their study, McKay et al. observed that the portions of meal kits delivered by two different suppliers did not meet the recommended amount of dietary fibre (21 g), according to the guidelines for pregnant women. The same observations were noted for magnesium (267 mg; 245 mg), calcium (522 mg; 339 mg), zinc (10 mg; 9 mg), iron (9 mg; 9 mg), vitamin B1 (1 mg; 1 mg), B2 (2 mg; 2 mg), and B12 (2 μg; 2 μg) [27]. In another study comparing the amount of nutrients provided by meal kits with the nutritional standards for the adult population, it was observed that no meal met the daily calcium requirement, while 50% of meals also failed to meet the magnesium requirement [26]. The protein content in all of the studies was kept within the dietary standards for the Australian and New Zealand populations (20.3–26.5% of energy). The excessively high fat content of the meal kit portions translated into am excessively low total carbohydrate content as compared with the dietary recommendations in relation to Australian standards (28–34% of energy) [24,25,26,27]. It bears noting that homemade equivalents of meal kits contained comparable amounts of nutrients to the ready-made kits [27].

**Table 1:** Energy and macronutrient content in one meal kit (average).

	Gibson and Partridge [24], (2019)	Moores et al. [25], (2020)	McKay [26], (2023)	McKay et al. [27], (2023)
Energy (kcal)	691.6–934.0	678.0	640.7–879.4	1224.0–1785.0
Protein (% of energy)	17.6–24.2	25.0	23.0–30.0	21.5–28.1
Fats (% of energy)	39.5–59.6	38.0	38.0–46.0	34.6–46.4
Carbohydrates (% of energy)	18.3–35.9	34.0	29.0	26.0–30.1

**Table 2:** Summary of diet quality.

	Study Type	Country	Sample Characteristic	Key Findings
Gibson and Partridge [24], (2019)	Descriptive research	Australia	60 meals from 12 randomly selected recipes (from 5 different companies)	High fat content (39.5 ± 9.5–59.6 ± 11.2% of energy) High sodium content (723 ± 404–1426 ± 688 mg per one meal serving) The energy content per serving ranged from 690.5 ± 128.7 to 932.5 ± 212.6 kcal Practically all the meals met the AI requirements for dietary fibre for both women and men and exceeded the level of SDT for women, while none met the high SDT level for men On average, all the meals covered 30% of the AI, RDI, and SDT for most nutrients for both men and women
Moores et al. [25], (2020)	Longitudinal	Australia	251 recipes for meal kits (249 recipes that have full data)	High fat content (35% of energy) and saturated fats (11% of energy) High sodium content (8% of respective meals exceeded the total recommended daily intake of sodium) The meal serving provided an average of 678 kcal (2840 kJ)
McKay [26], (2023)	Descriptive research	Australia	36 meal kits	High fat content (38–46% of energy) High sodium content (30% of the reference value set out in the Australian guidelines) The meals did not meet the calcium content requirements in the diet, and 50% also failed to meet the magnesium content requirements
McKay et al. [27], (2023)	Descriptive research	Australia	36 meal kits	High sodium content Low contents of fibre, magnesium, calcium, zinc, iron, vitamins B1, B2, and B12
Choi et al. [28], (2024)	Qualitative research	Australia	21 meal kits	The contents of energy, macronutrients, saturated fatty acids, trans fats, and sodium did not differ significantly from those in home-cooked meals A higher sugar content compared with that in home-cooked meals The sodium content in meal portions exceeded the recommended values

AI—Adequate intake; RDI—Recommended dietary intake; SDT—Suggested dietary target.

### 3.2. Acceptability of Meal Kits

Questionnaire surveys assessing the acceptability of meal kits by consumers demonstrated a significant satisfaction with that type of nutrition [32,33,34,35,36,37,38]. Meal kit subscribers emphasised that one of the most important benefits of ordering the kits was the reduction in time spent preparing meals [32,35,37]. Participants in the study conducted by Oberle et al. declared that it took them between 10 and 15 min to prepare meals [35]. In another pilot study conducted by Robinson-Ogjogho in the United States, subscribers reported a significant reduction in time spent preparing meals during the day, which also translated into experiencing pleasure in cooking healthy meals [32]. Some respondents stated that meal kits made it easier for them to select appropriate portion sizes and to consume meals regularly, which had previously been a significant problem for them [35,38]. An intervention among patients with pre-diabetes showed that knowledge about the portion sizes they should consume reduced the stress associated with it and allowed them to regain control over the amount of food they consumed [38]. Another significant advantage of meal kits was considered to be the expansion of knowledge of healthy food and the development of culinary skills [37,39]. The study conducted by Horning found that subscribing to meal kits was associated with the discovery of new food products and their incorporation into previously unfamiliar dishes. Additionally, meal kit use facilitated the development of culinary skills. Some participants also reported satisfaction with their newfound ability to season dishes and create various spice blends [35]. Research shows that individuals who have begun using meal kit services consume fruit and vegetables more frequently than they did before subscribing [32,33,39]. In a study conducted with 1413 participants during the COVID-19 pandemic, it was found that new meal kit subscribers consumed more fruit and vegetables (PR: 1.95, 95% CI: 1.42–2.68), but they also increased their intake of red and processed meats (PR: 2.39, 95% CI: 1.49–3.85) as compared with the period before subscribing [33]. Meal kits were characteristic not only of their ease and speed of preparation, but also their sensory appeal [34,35,37]. Respondents participating in the study conducted by Robinson-Oghogho reported their satisfaction with meals that included poultry, beef, and seafood. However, they rated vegetarian dishes slightly lower [34]. Despite that, the majority of respondents stated that they would have been willing to continue their meal kit subscriptions and would have recommended them to friends [34,36]. The prices of the meal kits, which were deemed affordable in relation to the quantity and quality of the meals received, undoubtedly had an impact on that as well [36,37]. The increased involvement of the entire family in the preparation of meal kits was also recognised as an advantage [39]. The issues reported by respondents included the amount of waste generated from the meal kit deliveries, the frequent repetition of meals in a short period of time, and the lack of freshness of some products [38]. Waste reduction could be facilitated through more precise portioning of ingredients required for meal preparation and consumer education on proper food storage to extend product freshness. Additionally, the implementation of reusable packaging, which consumers could return with subsequent deliveries, should be considered. Further strategies include the use of biodegradable materials or packaging designed for recycling. The characteristics of the included studies are summarised in Table 3.

**Table 3:** Summary of customer acceptability.

	Study Type	Country	Sample Characteristic	Key Findings
Robinson-Oghogho et al. [32], (2023)	Pilot study	United States	23 SouthEats consumers, from 26 to 69 years	Subscribing to meal kits decreased the time spent on meal preparation, increased the sense of healthy cooking, and resulted in more frequent consumption of fruit and vegetables
Robinson-Oghogho et al. [33], (2022)	Implementation research	United States	1413 participants, the majority of users were under the age of 55 (92.5%)	Meal kits contributed to the increased consumption of vegetables, fruit, as well as red and processed meat
Robinson-Oghogho et al. [34], (2023)	Cross-sectional	United States	35 SouthEats consumers and 3 employees, average age 42 years	Participants declared themselves to have been satisfied with the taste and visual appeal of the poultry, beef, and seafood dishes; the vegetarian dishes were rated slightly lower. Most users reported that they would have liked to continue subscribing to the meal kits
Oberle et al. [35], (2020)	Pilot study	United States	8 adults and 4 teenagers (average age 12.7 years)	Meal kits were characterised by their ease and speed of preparation. They made it easy to select appropriate meal portions and the accompanying lists of essential products reduced stress when shopping. Some participants felt that the meals could have been more flavoursome.
Horning et al. [36], (2021)	Pilot study	United States	230 participants	Participants felt that the meal kits were healthy and affordable, and almost all of the participants declared that they would have recommended them to others. The meal kits were found to have developed cooking skills, encouraged the preparation of new dishes, and contributed to the increased use of spices
Lee et al. [37], (2021)	Cross-sectional	Korea	404 participants, from 20 to 39 years	Meal kits were found to be convenient, reduce meal preparation time, and have a wide menu. Satisfaction with the freshness of the ingredients, their quantity, the taste of the meals, and the price was also observed.
Conroy et al. [38], (2024)	Qualitative research	New Zealand	7 participants, from 45 to 64 years	Meal kits made it easier to control meal size, regularity, and provided knowledge of healthy food and proper eating habits. The problems included the amount of waste, the appearance of stale products, and the repetitiveness of the kits.
Fraser et al. [39], (2022)	Evaluative research	Australia	16 women, from 30 to 48 years	Meal kits decreased the stress associated with making food-related decisions, increased the involvement of the entire family in meal preparation, expanded their knowledge of healthy eating, increased the consumption of fruit and vegetables, and decreased the intake of processed and take-away food.

### 3.3. Food Safety

**Figure 2:**
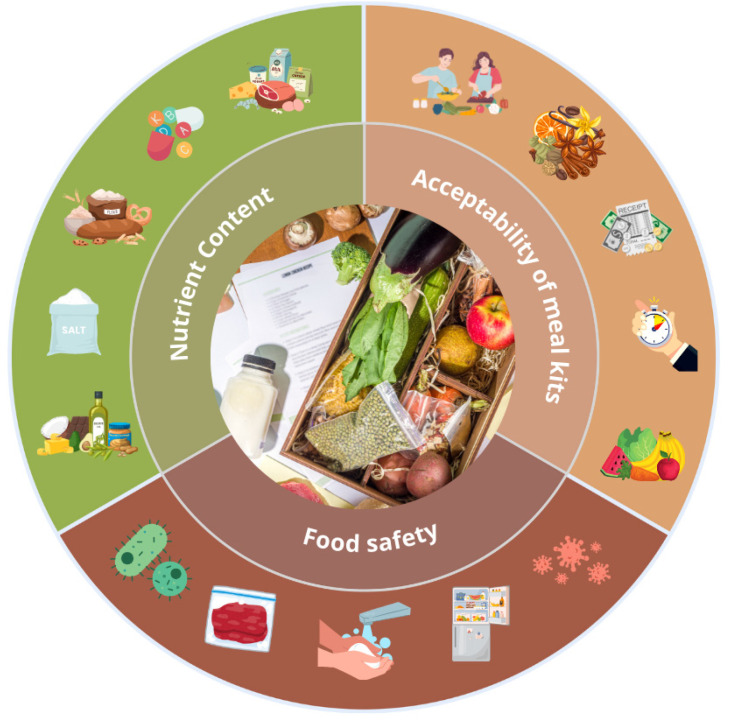
Factors that affect food quality.

The quality of food is determined not only by its nutritional value and organoleptic quality, but also by food safety (Figure 2). Food safety is crucial for public health [40]. The study conducted by Melville et al. [41] on food safety in 359 meal kit recipes delivered in the UK found that producers did not provide adequate information to mitigate the risk of food spoilage. A significant proportion of consumers did not receive guidance on appropriate storage conditions for the delivered food, potentially increasing the risk of pathogen proliferation. Only 50% of the recipe cards advised storing products in a refrigerator, and fewer than 1% specified a storage temperature below 5 °C. Most recipe cards (88%) included instructions to wash fruit and vegetables, while recommendations to wash herbs were present in 51%. Advice on handwashing before meal preparation was included in 46% of the cards, whereas 48% recommended washing hands during meal preparation. However, none of the cards provided instructions on proper handwashing techniques or emphasised the importance of drying hands afterwards. Guidelines to prevent cross-contamination were present in 51% of the recipe cards, typically recommending the use of separate cutting boards and kitchen utensils, as well as washing equipment between uses. Additionally, 98% of the recipes included cooking-related instructions. The most common information referred to visual cues, such as colour changes in cooked animal proteins (36%), cooking time in minutes (26%), and total or complete cooking time (16%). However, fewer than 1% of the cards mentioned the use of a meat thermometer [41]. The study conducted by Lee et al. [42] evaluated the delivery conditions and microbiological contamination of meal kits. The analysis included five types of mille-feuille nabe containing beef, pork, bean sprouts, various types of cabbage, mushrooms, and sauces, as well as three types of spring rolls made with rice paper, vegetables, and sauce. The average surface temperature of the products at the time of delivery was 14.4 °C, exceeding the Food Code guidelines, which recommend that chilled food be stored and transported at temperatures between 0 °C and 10 °C. Exceeding the recommended temperature promotes the growth of bacteria such as *Salmonella* spp., *E. coli*, and *Listeria monocytogenes*, the presence of which may lead to foodborne illnesses. Higher temperatures reduce product quality by causing losses of freshness, taste, and nutritional value. Additionally, products stored under improper conditions have a significantly shorter shelf life. The observed temperature exceedance may have resulted from elevated temperatures inside delivery vehicles and prolonged transport times for the meal kits. Despite this, the aerobic bacterial content in the meat remained within the limits set by the Guidelines for Microbiological Testing of Meat issued by the Ministry of Agriculture, Food, and Rural Affairs. For vegetables, the standards outlined by the Public Health Laboratory Service (PHLS) were applied. While most samples complied with these criteria, minor exceedances were noted in mushrooms and onions. The presence of Escherichia coli bacteria was also found in some vegetables, at levels that approached the upper tolerance limit for those microorganisms. No *Salmonella* spp. or *E. coli* O157 bacteria were found in any of the products; however, the presence of *Listeria monocytogenes* was confirmed in all the delivered portions of beef [42]. The characteristics of the included studies are summarised in Table 4.

**Table 4:** Summary of food safety of meal kits.

	Study Type	Country	Sample Characteristic	Key Findings
Melville et al. [41], (2023)	Descriptive research	The United Kingdom	359 recipe cards	Only 50% of the recipe cards included information on the necessity of storing the delivered products in the refrigerator, out of which <1% referred to storage at a temperature below 5 °C. Most cards contained information on the necessity of washing vegetables, fruit, and herbs. Additionally, 46% of the cards recommended to wash hands before cooking, while 48% suggested doing so during the cooking process. The majority of recipes (98%) included cooking-related statements in the meal preparation instructions.
Lee et al. [42], (2022)	Cross-sectional	Korea	5 sets of mille-feuille nabe and 3 sets of spring rolls	The average surface temperature of the products at the time of delivery was 14.4 °C, which exceeded the guidelines of the Food Code. The level of aerobic bacteria in the meat was kept within the acceptable range, while it was slightly exceeded in the case of vegetables. No presence of *Salmonella* spp. or *E. coli* O157 was observed in the tested products; however, the presence of *Listeria monocytogenes* was found in all the portions of beef.

## 4. Discussion

The findings of this review indicate a high potential for meal kits to be used for nutrition purposes as far as the prevention of diet-related diseases is concerned. With appropriately composed recipes, meal kits may become a much healthier alternative to take-away meals, while also providing consumers with all the essential nutrients. However, changes should be made to reduce the contents of fat and salt, which significantly exceed the guidelines [24,25,26,27,28]. It also seems important to refrain from recommending that consumers add products that are sources of fat and salt to dishes on the recipe cards [24]. Despite that, it is worth noting that meal kit subscribers report a higher consumption of fruit and vegetables than in the period before they started purchasing them [33,35].

The aforementioned review noted significant consumer satisfaction with meal kit subscriptions. Some respondents expressed a desire to continue that form of nutrition. They stated that meal kit delivery services had allowed them to reduce the time spent on cooking, facilitated the adjustment of meal portions and their regular consumption, decreased the stress associated with shopping, and expanded their knowledge of healthy foods and culinary techniques [33,34,35,36,37,38,39]. The study conducted by Fraser suggests that meal kit subscriptions could also expand parents’ knowledge of healthy eating, which, in turn, would facilitate nutritional education for their children [43]. Nutritional education for children and adolescents plays a vital role in fostering healthy eating habits and overall well-being. Evidence from an interventional study indicates that a comprehensive educational program for teenagers led to increased knowledge about nutrition, improved dietary behaviour, and enhanced health outcomes. Therefore, adopting a multidisciplinary approach to nutritional education is essential. This approach should integrate both theoretical and practical components, actively engaging young people in learning about nutrients, cooking techniques, and informed food choices. Such educational strategies can contribute to the development of sustainable healthy eating habits. Furthermore, introducing nutritional education at an early age can play a significant role in preventing diet-related diseases, including obesity, type 2 diabetes, and cardiovascular conditions [44]. The related literature data indicate that subscribers, when choosing that type of meal plan, pay particular attention to the availability of information on the origin of ingredients and the production of meal kits, ease of preparation, and price [45,46,47,48,49]. Respondents also reported increased consumption of fruit and vegetables since adopting meal kit services. Fraser analysed 179 recipes from companies operating in Australia and found that more than half of the examined meal kits provided at least half (2.7 servings) of the daily recommended vegetable intake for adults. However, the vegetable content varied across different meal kits. These findings suggest that meal kits have the potential to support higher vegetable consumption, particularly for families who select recipes with a greater quantity and variety of vegetables [50]. Although meal kit subscriptions seem quick and easy, there are also barriers that discourage some potential customers. In the study conducted by Chiong, respondents expressed concerns about orders online, the costs associated with that type of meal service, the lack of delivery options outside urban areas, and uncertainties regarding the quantity and quality of the products [51]. The aforementioned barriers may stem from a lack of sufficient knowledge of meal kits. The authors highlight the importance of reaching consumers living in rural areas and older individuals to raise awareness about the benefits of meal kit subscriptions. The literature data gathered in this review indicate insufficient food safety offered by the companies. It seems crucial to enhance the recipe cards with information on food storage, handling, and adherence to hygiene practices during meal preparation [41,42]. Recipe cards should primarily include information on the appropriate storage temperatures for food from different product groups, as well as how long they can be stored. It would be helpful to enrich the cards with notes on cooking techniques and necessary equipment, which could facilitate meal preparation. It also seems essential to emphasise the importance of basic hygiene practices, such as washing hands, food items, and kitchen utensils, as this would significantly reduce the risk of foodborne infections. Producers should also make greater efforts to adhere to the timing and conditions of meal kit deliveries, which would eliminate the possibility of food spoiling quickly [42]. Research shows that meal kits could serve as a healthy alternative to traditional diets. However, improvements in the quality of the kits offered by companies are necessary. Ready-to-use products with accompanying recipes may assist individuals who lack the time to prepare balanced meals and those who may not have knowledge of healthy eating. Meal kits could also be beneficial in the nutritional treatment of patients requiring specific macronutrient delivery or the exclusion of certain foods from their diets. Further research using analytical methods to validate the nutritional data and assess the nutritional content of meal kits appears essential.

This review has several strengths, as well as certain limitations. A key strength of this study is the comprehensive synthesis of reliable information on meal kits, addressing critical aspects such as nutritional content, consumer acceptability, and safety. To the authors’ knowledge, this is the first scoping review to examine all these factors collectively. Additionally, the gathered findings are relatively consistent across multiple publications, further supporting this study’s robustness. However, some limitations should be acknowledged. The small number of studies on meal kits may affect the reliability of their evaluation. Moreover, there is a notable lack of research utilising analytical methods to assess the nutritional content of meal kits. While all existing studies have employed computational methods, analytical techniques offer more precise measurements by directly quantifying nutrient levels in food. Unlike computational methods, which rely on general nutrient data and outdated databases, analytical approaches minimise the risk of errors and account for the complexity of food composition. Future research employing such methods could yield more accurate and reliable results. Another limitation is the geographical focus of existing studies, which are predominantly conducted in Australia and the United States. The absence of research from other regions limits the generalisability of the findings, highlighting the need for further investigations in diverse geographical contexts.

## 5. Conclusions

Meal kits may serve as a practical nutritional solution for individuals with limited time for meal preparation or insufficient cooking skills. When properly balanced, these kits could contribute to the prevention of lifestyle-related diseases, many of which are closely linked to poor dietary habits. This review included studies examining the nutrient content, product quality, and consumer acceptability of home-delivered meal kits. The exclusion criteria encompassed review papers, systematic reviews without meta-analyses, and studies published more than 10 years ago. The findings of this review may offer valuable insights for meal kit manufacturers, enabling them to enhance the nutritional quality of their products to ensure that they provide consumers with essential nutrients in appropriate proportions. However, it is important to acknowledge that the available data on the nutritional value of meal kits primarily originate from studies employing computational methods, which may have led to reduced accuracy in the reported results. Therefore, more detailed analytical research is needed to provide precise measurements of actual nutrient content. Additionally, intervention studies assessing the effects of meal kit-based diets on consumers’ health parameters would be beneficial. The findings of this review highlight the considerable potential of meal kits in promoting healthier dietary patterns. Improving the nutritional quality of meal kit services could contribute to better overall public health outcomes.

## Data Availability

All information will be available upon an e-mail request to the corresponding author.
